# Characteristics of and Deaths among 333 Persons with Tuberculosis and COVID-19 in Cross-Sectional Sample from 25 Jurisdictions, United States

**DOI:** 10.3201/eid2910.230286

**Published:** 2023-10

**Authors:** Scott A. Nabity, Suzanne M. Marks, Neela D. Goswami, Shona R. Smith, Evan Timme, Sandy F. Price, Lon Gross, Julie L. Self, Katelynne Gardner Toren, Masahiro Narita, Donna H. Wegener, Shu-Hua Wang, for the National Tuberculosis Controllers Association/CDC TB-COVID-19 Collaboration

**Affiliations:** California Department of Public Health, Richmond, California, USA (S.A. Nabity);; Centers for Disease Control and Prevention, Atlanta, USA (S.A. Nabity, S.M. Marks, N.D. Goswami, S.F. Price, L. Gross, J.L. Self);; Michigan Department of Health and Human Services, Lansing, Michigan, USA (S.R. Smith);; Arizona Department of Health Services, Phoenix, Arizona, USA (E. Timme);; Public Health–Seattle & King County, Seattle, Washington, USA (K. Gardner Toren, M. Narita);; University of Washington, Seattle (M. Narita);; National Tuberculosis Controllers Association, Atlanta (D.H. Wegener);; The Ohio State University College of Medicine, Columbus, Ohio, USA (S.-H. Wang)

**Keywords:** COVID-19, tuberculosis and other mycobacteria, coronavirus disease, SARS-CoV-2, severe acute respiratory syndrome coronavirus 2, viruses, bacteria, bacterial infection, respiratory infections, zoonoses, vaccine-preventable diseases, United States

## Abstract

Little is known about co-occurring tuberculosis (TB) and COVID-19 in low TB incidence settings. We obtained a cross-section of 333 persons in the United States co-diagnosed with TB and COVID-19 within 180 days and compared them to 4,433 persons with TB only in 2020 and 18,898 persons with TB during 2017‒2019. Across both comparison groups, a higher proportion of persons with TB–COVID-19 were Hispanic, were long-term care facility residents, and had diabetes. When adjusted for age, underlying conditions, and TB severity, COVID-19 co-infection was not statistically associated with death compared with TB infection only in 2020 (adjusted prevalence ratio 1.0 [95% CI 0.8‒1.4]). Among TB–COVID-19 patients, death was associated with a shorter interval between TB and COVID-19 diagnoses, older age, and being immunocompromised (non-HIV). TB–COVID-19 deaths in the United States appear to be concentrated in subgroups sharing characteristics known to increase risk for death from either disease alone.

Tuberculosis (TB) and COVID-19 were leading infectious causes of illness and death globally in 2020. In the United States, >17 million COVID-19 cases and ≈7,000 TB cases were reported in 2020 (*1*). Both TB and COVID-19 are primarily respiratory illnesses with overlapping signs and symptoms, and the Centers for Disease Control and Prevention (CDC) lists TB as a medical risk factor for COVID-19‒related disease severity and death (*2*). Few population-based reports of outcomes for persons with both TB and COVID-19 have been reported (*3*,*4*), and the definition of TB and COVID-19 co-diagnosis differs across studies. Those reports, and meta-analyses incorporating case reports and small observational series (*5*–*8*), have demonstrated higher mortality rates for persons with TB and COVID-19 compared with COVID-19 alone. Little has been published to adequately assess COVID-19 as a risk factor for poor TB outcomes. Furthermore, limited information is available from low TB incidence populations, including the United States. An analysis from California showed increased mortality rates for persons with TB and COVID-19 compared with TB only reported before the COVID-19 pandemic, particularly when TB and COVID-19 were diagnosed in close succession (*9*). That analysis indicated groups of persons who were disproportionately affected by both diseases, including Hispanic persons and those with diabetes or living in low health equity neighborhoods according to the California Healthy Places Index (*10*).

The COVID-19 pandemic also affected TB epidemiology and program management across epidemiologic contexts (*11*). In the United States, reported TB incidence declined ≈20% in 2020 compared with 2019 (*12*). Limited information suggests that some persons with TB in the United States may have had more clinically severe disease in 2020 than before the COVID-19 pandemic (*13*), and TB diagnoses may have been delayed (*14*). We aimed to describe demographic, social, and clinical characteristics of persons with TB and COVID-19 in the United States, including risk for death, and to identify populations who may benefit most from integrated interventions.

## Materials and Methods

### Design and Population

We established a voluntary collaboration of US health jurisdictions to obtain a cross-sectional sample of persons with TB and COVID-19 diagnosed within 180 days (hereafter TB–COVID-19). We used the population-based National Tuberculosis Surveillance System (NTSS) for cases reported during 2017‒2021 for standardized demographic, social, underlying conditions, and TB-specific diagnosis and treatment variables (*15*). Each jurisdiction captured a subset of the standardized data elements from the National Notifiable Disease Surveillance System for COVID-19 cases (*16*) and contributed them to this project. The core set of COVID-19 and TB surveillance data elements were consistent across jurisdictions. We included all persons with COVID-19 meeting the public health surveillance case definition for confirmed or probable COVID-19 (*17*) reported during January 1, 2020–June 30, 2021, who were also persons with TB reported in 2020 (i.e., persons with TB–COVID-19). Although the methods used by participating jurisdictions to identify persons with TB–COVID-19 varied (Appendix Table 1), each jurisdiction systematically identified their residents with co-diagnoses of TB and COVID-19 using personal identifiers. Of 26 participating jurisdictions, 11 (42.3%) used a software algorithm that included name and date of birth to match persons (several also included various combinations of sex, race/ethnicity, and place of residence), 8 (30.8%) had integrated surveillance systems (i.e., a given individual’s TB and COVID-19 diagnoses were already linked to a single record), and 1 (3.8%) with an integrated surveillance system also performed a name-based software match (Appendix Table 1). Directly identifiable information in the surveillance registries was retained by participating jurisdictions and not shared with investigators in other jurisdictions or with CDC.

**Table 1:** Characteristics of 333 persons from 25 US jurisdictions who were diagnosed with TB–COVID-19 in 2020 and persons with TB only diagnosed in 2017‒2019 or in 2020*

Characteristic	2020 TB–COVID-19		2020 TB only			2017‒2019 TB only	
			Value	p value		Value	p value
Total no.	333		4,433			18,898	
Median age at TB diagnosis, y (IQR)	55 (35‒69)		51 (32‒66)	0.0834		50 (32‒66)	0.0564
0‒4	<5		104 (2.3)	0.0373		435 (2.3)	0.0389
5‒14	6 (1.8)		92 (2.1)	0.7345		360 (1.9)	0.8914
15‒24	33 (9.9)		440 (9.9)	0.9927		1,778 (9.4)	0.7561
25‒44	82 (24.6)		1,220 (27.5)	0.2527		5,525 (29.2)	0.0720
45‒64	106 (31.8)		1,341 (30.2)	0.5450		5,700 (30.2)	0.5105
65‒74	53 (15.9)		630 (14.2)	0.3920		2,469 (13.1)	0.1266
75‒85	39 (11.7)		416 (9.4)	0.1634		1,842 (9.8)	0.2316
>85	12 (3.6)		190 (4.3)	0.5511		807 (4.3)	0.5503
Sex							
M	204 (61.3)		2,707 (61.1)	0.9435		11,602 (61.4)	0.9601
F	129 (38.7)		1,726 (38.9)			7,296 (38.6)	
Race/ethnicity							
Missing/unknown	2		13			61	
White	26 (7.8)		445 (10.1)	0.1939		1,993 (10.6)	0.1094
Black	49 (14.8)		787 (17.8)	0.1666		3,201 (17.0)	0.2926
Asian	101 (30.5)		1,691 (38.3)	0.0051		7,047 (37.4)	0.0101
Hispanic	146 (44.1)		1,433 (32.4)†	<0.0001		6,278 (33.3)‡	<0.0001
NHOPI	5 (1.5)		25 (0.6)	0.0363		128 (0.7)	0.0710
American Indian/Alaska Native	<5		13 (0.3)	0.0072		99 (0.5)	0.0921
Multiple	0		26 (0.6)			91 (0.5)	
Non–US-born	264 (79.3)		3,195 (72.3)	0.0059		13,645 (72.2)	0.0045
Missing/Unknown	0		15			12	
Years in United States, non–US born	17 (7‒27)		12 (3‒28)	0.0031		12 (2‒26)‡	0.0005
Long-term care facility resident at time of TB diagnosis§	15 (4.6)		68 (1.6)†	<0.0001		341 (1.9)‡	0.0004
Missing/unknown	10		207			822	
Correctional facility resident at time of TB diagnosis¶	11 (3.4)		137 (3.3)	0.8876		694 (3.8)	0.6739
Missing/unknown	9		218			864	
Homeless within year before TB diagnosis§	12 (3.7)		179 (4.2)	0.6549		826 (4.6)	0.4638
Missing/unknown	11		217			891	
Excessive alcohol within year before TB diagnosis§	24 (7.6)		366 (8.8)	0.4692		1,488 (8.3)	0.6349
Missing/unknown	17		267			1,055	
Drug use within year before TB diagnosis¶	14 (4.4)		322 (7.7)	0.0332		1,384 (7.8)	0.0293
Missing/Unknown	18		262			1,029	
Employed§	123 (74.5)		1,623 (66.6)	0.0346		7,604 (67.5)	0.0540
Missing/unknown	49		365			504	
Healthcare worker§	15 (5.2)		144 (3.5)	0.1007		631 (3.4)	0.0538
Missing/unknown	47		365			504	
Previous episode of TB	11 (3.4)		215 (4.9)	0.2135		872 (4.6)	0.2753
Missing/unknown	6		32			110	
Site of TB disease							
Missing/unknown	0		0			5	
Pulmonary only	223 (67.0)		2,997 (67.6)	0.8100		12,950 (68.5)	0.5391
Extrapulmonary only	69 (20.7)		874 (19.7)	0.6571		3,749 (19.8)	0.6907
Both	41 (12.3)		562 (12.7)	0.8467		2,194 (11.6)	0.6930
HIV-positive at TB diagnosis	14 (4.7)		186 (4.8)	0.9740		811 (4.9)	0.9011
Missing/unknown	37		535			2,302	
Other immunocompromising condition#	27 (8.1)		285 (6.4)	0.2323		998 (5.3)	0.0228
Diabetes	122 (36.6)		1,044 (23.6)†	<0.0001		4,138 (21.9)‡	<0.0001
End-stage renal disease	21 (6.3)		155 (3.5)	0.0087		544 (2.9)‡	0.0002
AFB sputum smear positive	155 (53.3)		1795 (46.9)	0.0373		7354 (45.2)	0.0061
Missing/unknown	42		609			2,623	
Sputum *Mycobacterium tuberculosis* complex culture–positive	206 (71.8)		2,566 (67.9)	0.1782		11,077 (69.6)	0.4360
Missing/unknown	46		657			3,003	
NAAT-positive	210 (82.4)		2,458 (74.1)	0.0034		9,648 (72.5)	0.0004
Missing/unknown	78		1,115			5,583	
Cavity on chest imaging**	119 (41.6)		1,395 (36.6)	0.0935		5,777 (35.6)	0.0350
Missing/unknown	47		626			2,663	
Disseminated TB††	60 (18.0)		717 (16.2)	0.3797		2,763 (14.6)	0.0925

*Values are no. (%) except as indicated. Percentages were calculated after subtracting missing and unknown responses. AFB, acid-fast bacilli; IQR, interquartile range; NAAT, nucleic acid amplification test; NHOPI, Native Hawaiian and other Pacific Islander; TB, tuberculosis; TB–COVID-19, diagnosed with both TB and COVID-19 within 180 days. †Statistically significant comparison between persons with TB–COVID-19 and 2020 TB-only with Bonferroni correction (critical value 0.0017). ‡Statistically significant comparison between persons with TB–COVID-19 and 2017‒2019 TB-only with Bonferroni correction (critical value 0.0017). §Among persons >15 years of age. ¶Among persons >15 years of age. Includes injecting and noninjecting drugs. #Non-HIV immunocompromising condition attributable to medical conditions, such as hematologic or reticuloendothelial malignancies (e.g., leukemia, Hodgkin’s lymphoma, carcinoma of the head or neck), or immunosuppressive therapy, such as prolonged use of high-dose adrenocorticosteriods (e.g., prednisone), but not including immunocompromising condition related to HIV infection. **Among persons with pulmonary TB having chest radiograph, chest computed tomography, or both. ††Defined as both pulmonary and extrapulmonary sites of disease, positive blood culture for *Mycobacterium tuberculosis* complex, meningitis, or miliary pattern on imaging.

**Figure 1:**
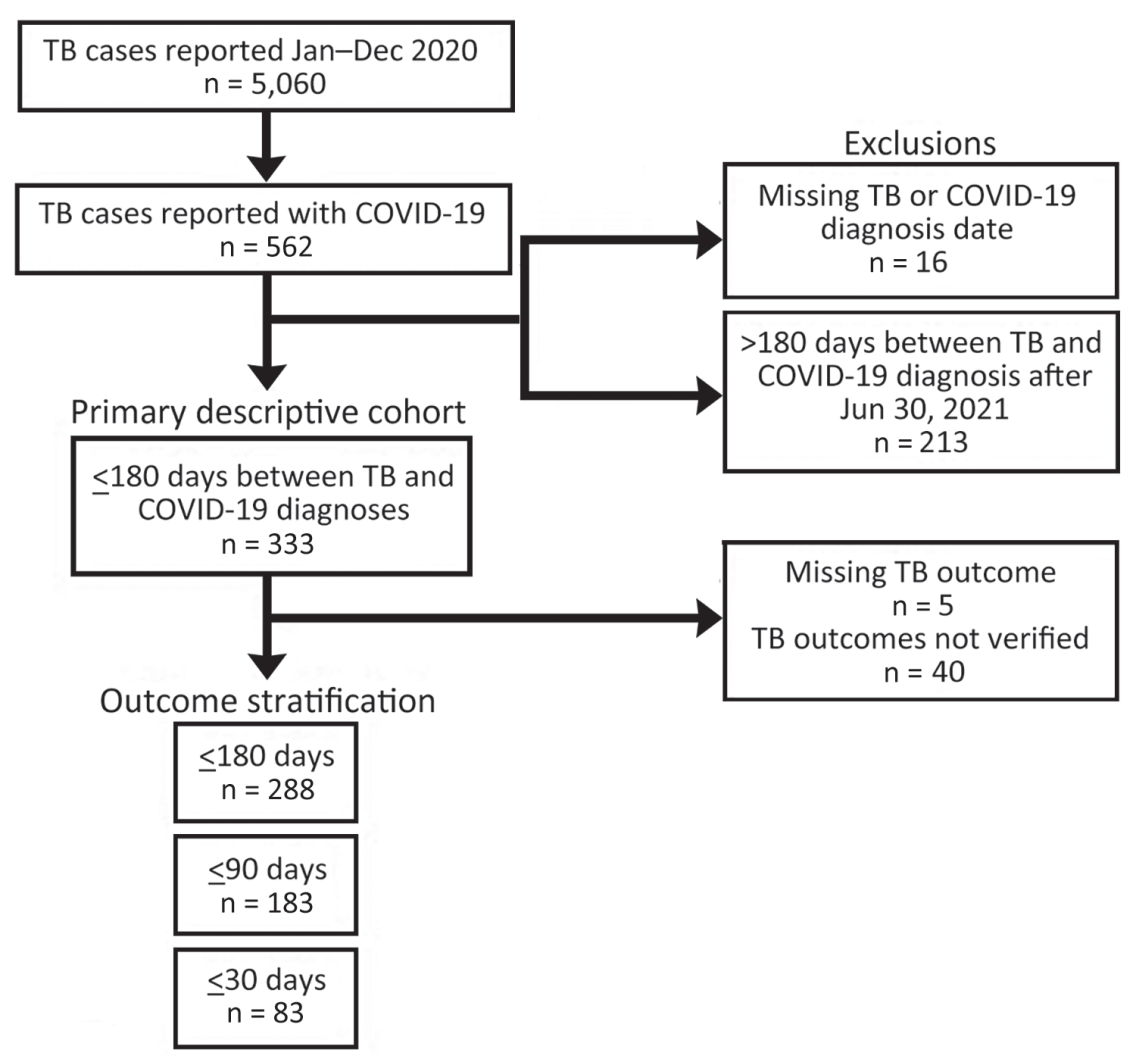
Analytic sample selection for persons with TB and COVID-19 co-diagnosed within 180 days (TB–COVID-19), 26 US jurisdictions, 2020. Three states performed registry matches with COVID-19 data up-to-date through an earlier date (January 24, 2021; February 2, 2021; August 31, 2021); 1 US state (North Dakota) that participated did not have TB–COVID-19 cases. The number of days between TB and COVID-19 diagnosis dates was calculated without regard for which disease was diagnosed first. Data from 2 jurisdictions (Puerto Rico and Los Angeles County; remainder of California included) were excluded because of incompleteness of outcomes data. TB, tuberculosis.

Each jurisdiction securely transmitted data on persons with TB–COVID-19 to CDC. We then excluded persons with TB–COVID-19 with unknown TB or COVID-19 diagnosis dates and for which diagnoses occurred >180 days apart, regardless of which disease was diagnosed first (Figure 1). For analysis of TB treatment outcomes, we excluded jurisdictions with incomplete TB case outcome data. To identify characteristics of persons with TB–COVID-19 that differed from persons with TB only, we compared them to characteristics of persons with TB reported in 2020 without COVID-19 (i.e., 2020 TB-only) and TB reported during the 3 most recent pre–COVID-19 years, 2017‒2019 (i.e., 2017‒2019 TB-only).

### Data Elements

NTSS data included demographic, social, and clinical characteristics, and TB diagnosis and treatment outcomes. We used a composite all-cause death outcome that included TB diagnosed after death, death occurring before or during TB treatment, and death recorded on the COVID-19 case report form. We defined the TB diagnosis date as the earliest among positive smear or tissue collection, positive nucleic acid amplification test result, first culture specimen collected for phenotypic drug-sensitivity testing, or TB treatment start date. For the COVID-19 diagnosis date, we used the date of specimen collection of the first positive nucleic acid amplification test or antigen test. We defined persons with disseminated TB as having meningeal or miliary disease, both pulmonary and extrapulmonary disease, or having a positive culture for *Mycobacterium tuberculosis* complex from blood.

### Analytic Methods

We compared characteristics of persons with TB–COVID-19 with those of persons with 2020 TB-only and 2017‒2019 TB-only, calculating statistically significant differences of bivariate frequencies by using the Mantel-Haenszel χ^2^ test (or Fisher exact test for small cell counts) with Bonferroni correction for multiple comparisons. We also calculated Clopper–Pearson binomial 95% CIs for some frequencies. For continuous variables, we assessed differences in parametric means by using t-tests. We used the Wilcoxon rank-sum test to compare nonparametric continuous variables. We calculated prevalence ratios (PRs) and 95% CIs by using log-binomial multivariable regression employing backward selection in logistic regression models to identify statistically significant (α = 0.05) variables for inclusion in the log-binomial models. The final models included all variables reaching statistical significance and the COVID-19 co-diagnosis status as the exposure of interest. We did not assess interaction terms in multivariable models. Rather than exclude persons with missing covariate data, we classified missing values as unknown and retained them in the models. We stratified outcomes by the proximity in timing of TB and COVID-19 diagnoses (i.e., within 30, 90, and 180 days) and fit independent log-binomial models to each time interval.

### Ethics Considerations

This activity was determined to meet the requirements of public health surveillance as defined in 45 CFR 46.102(l) (*2*). Informed consent was not required because the project was classified by CDC as nonresearch. Although most participating jurisdictions relied on the CDC project determination, some independently classified the activity as nonresearch.

## Results

### TB–COVID-19 Analytic Population

The 26 participating jurisdictions accounted for 62.9% of US TB cases in 2020 and 67.0% of the 2020 US population (*18*). The number of all TB cases reported in 2020 per participating jurisdiction ranged from 10 to 1,703: 12 jurisdictions (46.2%) reported <75 cases, 8 (30.8%) reported 75‒149 cases, and 6 (23.1%) reported >150 cases. Participating jurisdictions reported ≈64% of the ≈46,353,000 COVID-19 cases reported in US states and territories reported during the observation period (*1*). Jurisdictions using more robust methods (i.e., a software algorithm or an integrated surveillance system) to identify persons with TB–COVID-19 (Appendix Table 1) accounted for 91.7% of the TB cases among the 26 participating jurisdictions.

**Figure 2:**
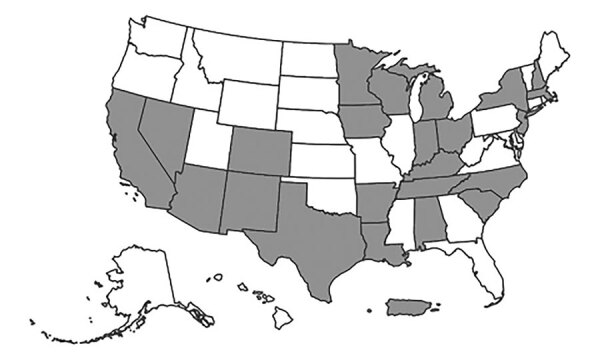
Locations of 25 US jurisdictions contributing data for 333 persons with tuberculosis (TB) and COVID-19 co-diagnosed within 180 days (TB–COVID-19), 2020. Participating jurisdictions: Alabama (n = 5 cases), Arizona (n = 21), Arkansas (n = 9), California (n = 114), Colorado (n = 7), Indiana (n = 10), Iowa (n = 2), Kentucky (n = 6), Louisiana (n = 3), Massachusetts (n = 7), Michigan (n = 11), Minnesota (n = 14), Nevada (n = 4), New Hampshire (n = 1), New Jersey (n = 28), New Mexico (n = 4), New York State (n = 6); New York, NY (reporting separately; n = 37), North Carolina (n = 11), Ohio (n = 6), Puerto Rico (n = 2), South Carolina (n = 2), Tennessee (n = 11), Texas (n = 6), and Wisconsin (n = 6). North Dakota provided data but had no TB–COVID-19 cases reported.

**Figure 3:**
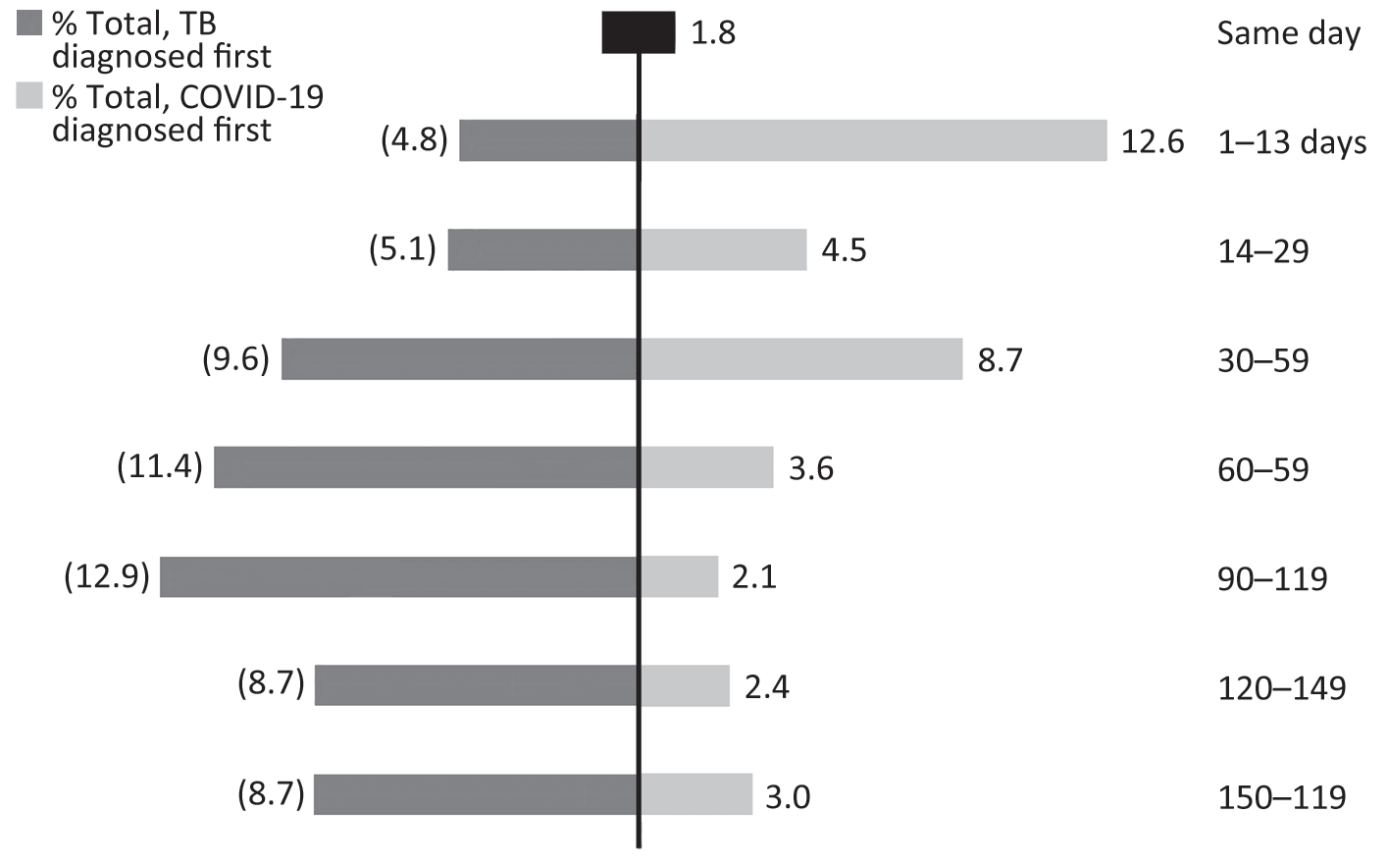
Frequency of 333 persons with TB and COVID-19 co-diagnosed (TB–COVID-19), by sequence and days between TB and COVID-19 diagnoses, 25 US jurisdictions, 2020. The percentage denominator accounts for all 333 persons. Individual percentages may not sum to 100% because of rounding. TB, tuberculosis.

One of 26 jurisdictions (North Dakota) did not identify persons with TB–COVID-19 meeting our criteria, and so we excluded numerator and denominator data for this jurisdiction from statistical analyses. The remaining 25 jurisdictions (23 US states; New York, NY; and Puerto Rico) identified 333 persons with TB–COVID-19 meeting study criteria (Figures 1, 2). The number of persons with TB–COVID-19 identified per health jurisdiction ranged from 1 to 114 (Figure 2). The median age of persons with TB–COVID-19 was 55 years (interquartile range 35‒69 years); 204 (61.3%) were male and 129 (38.7%) female, and 264 (79.3%) were non–US-born (Table 1). Six (1.8%) persons were co-diagnosed with TB and COVID-19 on the same date, and 65 (19.5%) persons were co-diagnosed within 14 days (Figure 3). Of the 327 TB–COVID-19 cases diagnosed >1 day apart, 204 (62.4%) had TB diagnosed before COVID-19.

### TB–COVID-19 Demographics Compared with 2020 TB-only and 2017‒2019 TB-only 

We did not find statistically significant (95% CI with Bonferroni correction) bivariate differences in persons with TB–COVID-19 relative to the 2020 TB-only and 2017‒2019 TB-only comparison groups for sex, residence in a correctional facility, homelessness, or excessive alcohol use (Table 1). The TB–COVID-19 group had a higher proportion of Hispanic persons compared with both of the reference groups (Table 1). Higher proportions of persons with TB–COVID-19 also were residents of long-term care facilities at TB diagnosis compared with both reference groups.

### TB–COVID-19 Clinical Characteristics Compared with 2020 TB-only and 2017‒2019 TB-only 

We did not find statistically significant differences, compared with either the 2020 TB-only or 2017‒2019 TB-only reference group, for the proportion of persons with TB–COVID-19 by the status of a previous episode of TB, HIV infection, TB disease site (i.e., pulmonary-only, extrapulmonary, or both sites), or TB disease dissemination (Table 1). Compared with both reference groups, persons with TB–COVID-19 had a higher rate of diabetes and end-stage renal disease.

### Multivariable Comparison of TB–COVID-19 Characteristics Compared with 2020 TB-only and 2017‒2019 TB-only

In comparison to 2020 TB-only, persons with TB and COVID-19 diagnosed within 180 days were more likely to be in American Indian/Alaska Native (adjusted prevalence ratio [aPR] 5.3 [95% CI 2.1‒13.4]) and, with borderline statistical significance, Native Hawaiian/Other Pacific Islander (aPR 2.3 [95% CI 1.0‒5.3]) relative to non-Hispanic whites (Table 2). Persons with TB–COVID-19 also had a higher proportion of being non–US-born (aPR 1.5 [95% CI 1.1‒2.1]), residing in long-term care facilities at TB diagnosis (aPR 2.5 [95% CI 1.6‒4.0]), and having diabetes (aPR 1.6 [95% CI 1.3‒2.0]). When comparing persons with TB–COVID-19 to 2017‒2019 TB-only, those associations remained statistically significant; in addition, a higher proportion of persons with TB–COVID-19 also had end-stage renal disease (aPR 1.7 [95% CI 1.1‒2.7]) and, with borderline statistical significance, acid-fast bacilli sputum smear positivity (aPR 1.3 [95% CI 1.0‒1.6]) (Table 2).

**Table 2:** Multivariable log-binomial regression of characteristics for 333 persons from 25 US jurisdictions who were diagnosed with TB–COVID-19 in 2020 and persons with TB only diagnosed in 2017‒2019 or in 2020*

Characteristic	2020 TB only			2017‒2019 TB only	
	uPR (95% CI)	aPR (95% CI)		uPR (95% CI)	aPR (95% CI)
Race/ethnicity†					
White	Referent	Referent		Referent	Referent
Black	0.8 (0.6‒1.1)	1.1 (0.7‒1.7)		0.9 (0.6‒1.1)	1.2 (0.7‒1.9)
Asian	0.7 (0.6‒0.9)	0.8 (0.5‒1.2)		0.7 (0.6‒0.9)	0.8 (0.5‒1.3)
Hispanic	1.6 (1.3‒2.0)	1.4 (0.9‒2.2)		1.6 (1.3‒1.9)	1.4 (0.9‒2.2)
NHOPI	2.4 (1.1‒5.4)	2.3 (1.0‒5.3)		2.2 (0.9‒5.2)	2.9 (1.1‒7.4)
American Indian/Alaska Native	3.4 (1.4‒8.1)	5.3 (2.1‒13.4)		2.3 (0.9‒6.0)	3.4 (1.2‒9.6)
Origin of birth					
US-born	Referent	Referent		Referent	Referent
Non–US-born	1.4 (1.1‒1.8)	1.5 (1.1‒2.1)		1.5 (1.1‒1.9)	1.6 (1.1‒2.2)
AFB sputum smear result					
Negative	Referent	NI		Referent	Referent
Positive	1.3 (1.0‒1.5)			1.4 (1.1‒1.7)	1.3 (1.0‒1.6)
Missing/Unknown	0.9 (0.7‒1.2)			0.9 (0.7‒1.2)	1.0 (0.7‒1.5)
Long-term care resident at TB diagnosis‡					
No	Referent	Referent		Referent	Referent
Yes	2.6 (1.6‒4.2)	2.5 (1.6‒4.0)		2.5 (1.5‒4.1)	2.4 (1.4‒4.0)
Diabetes					
No	Referent	Referent		Referent	Referent
Yes	1.8 (1.4‒2.2)	1.6 (1.3‒2.0)		2.0 (1.6‒2.5)	1.8 (1.4‒2.2)
End-stage renal disease					
No	Referent	NI		Referent	Referent
Yes	1.8 (1.2‒2.7)			2.2 (1.4‒3.4)	1.7 (1.1‒2.7)

*Model adjusted for other variables in the table. aPR, adjusted prevalence ratio; AFB, acid-fast bacilli; NHOPI, Native Hawaiian and Other Pacific Islander; NI, not included in adjusted regression model because not statistically significant at 95% confidence level; TB, tuberculosis; TB–COVID-19, diagnosed with both TB and COVID-19 within 180 days; uPR, unadjusted prevalence ratio. †Multiple race category not included because no persons with TB–COVID-19 reported multiple races. ‡Among persons >15 years of age.

### Mortality Rates Compared with 2020 TB-only Patients

**Figure 4:**
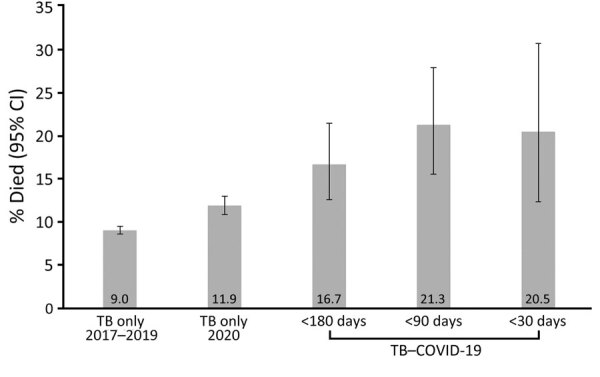
Unadjusted mortality rates for persons with TB only during 2017‒2019 and during 2020 compared with persons with TB–COVID-19 during 2020, 25 US jurisdictions. Error bars indicate 95% CIs. TB, tuberculosis; TB–COVID-19, diagnosed with both TB and COVID-19 within 180 days.

The occurrence of death at any time before or during TB treatment was 450/3,793 (11.9% [95% CI 10.8‒12.9]) for 2020 TB-only. This rate compares with 48/288 (16.7% [95% CI 12.6‒21.5]; unadjusted prevalence ratio [uPR] 1.4 [95% CI 1.1‒1.8]) for persons co-diagnosed with TB and COVID-19 within 180 days, 39/183 (21.3% [95% CI 15.6‒28.0]; uPR 1.8 [95% CI 1.3‒2.5]) for those co-diagnosed within 90 days, and 17/83 (20.5% [95% CI 12.4‒30.8]; uPR 1.7 [95% CI 1.1‒2.7]) for those co-diagnosed within 30 days (Figure 4). After adjustment for age, comorbidities, and markers of TB disease severity, COVID-19 did not retain significance as an independent risk factor for all-cause mortality in persons with TB disease (Appendix Table 2). Significant cofactors were age ≥45 years, HIV infection (aPR 2.1 [95% CI 1.3‒3.5]), end-stage renal disease (aPR 1.8 [95% CI 1.4‒2.4]), TB disease dissemination (aPR 1.5 [95% CI 1.1‒1.9]), and sputum smear positivity for acid-fast bacilli (aPR 1.4 [95% CI 1.1‒1.8]). We observed similar associations when persons had TB and COVID-19 diagnosed within shorter intervals (i.e., 30 and 90 days) (Appendix Table 2).

### Risk Factors for Death among TB–COVID-19 Patients

Among the 288 persons with TB–COVID-19 with known mortality outcome (86.5% of all 333 persons with TB–COVID-19), death was associated with both TB and COVID-19 co-diagnoses being made within 90 days (aPR 2.3 [95% CI 1.1‒4.8]), being immunocompromised (non-HIV) (aPR 2.7 [95% CI 1.1‒6.4]), and age (Table 3). The adjusted risk for death increased with increasing age compared with <44 years: 45‒64 years, aPR 5.6 (95% CI 1.6‒19.8); 65‒74 years, aPR 8.6 (95% CI 2.4‒31.3); 75‒84 years, aPR 12.6 (95% CI 3.5‒45.7); and >85 years, aPR 25.0 (95% CI 6.9‒91.1).

**Table 3:** Adjusted log-binomial risk factors associated with 48 deaths among 288 persons with TB–COVID-19 in 2020, 24 US jurisdictions*

Characteristic	No. (%) deaths‡	uPR (95% CI)	aPR (95% CI)
Timing of TB and COVID-19 diagnoses†			
>90 d	9 (8.6)	Referent	Referent
<90 d	39 (21.3)	2.5 (1.3‒4.9)	2.3 (1.1‒4.8)
Age at TB diagnosis, y			
<44	<5	Referent	Referent
45‒64	16 (18.0)	6.7 (2.0‒22.3)	5.6 (1.6‒19.8)
65‒74	11 (23.4)	8.7 (2.6‒29.9)	8.6 (2.4‒31.3)
75‒85	13 (40.6)	15.2 (4.6‒50.0)	12.6 (3.5‒45.7)
>85	5 (62.5)	23.3 (6.8‒80.5)	25.0 (6.9‒91.1)
Immunocompromising condition, non-HIV§			
No	39 (14.6)	Referent	Referent
Yes	9 (45.0)	3.1 (1.8‒5.4)	2.7 (1.1‒6.4)

*Model adjusted for other variables in the table. NTSS outcomes updated as of September 23, 2022. Five with missing outcome; there were no missing values for days between diagnoses, age, or immunocompromising condition (non-HIV). Puerto Rico and Los Angeles County excluded (remainder of California included) because of incompleteness of outcomes data. aPR, adjusted prevalence ratio; TB, tuberculosis; TB–COVID-19, diagnosed with both TB and COVID-19 within 180 days; uPR, unadjusted prevalence ratio. †Days between TB diagnosis and COVID-19 diagnosis, regardless of which was diagnosed first. †Percentage is the number of persons with TB–COVID-19 with the characteristic who died divided by the total number of persons with TB–COVID-19 with that characteristic. §Non-HIV immunocompromising condition attributable to medical conditions, such as hematologic or reticuloendothelial malignancies (e.g., leukemia, Hodgkin’s lymphoma, carcinoma of the head or neck), or immunosuppressive therapy, such as prolonged use of high-dose adrenocorticosteriods (e.g., prednisone), but not including immunocompromising condition related to HIV infection.

## Discussion

We report a large cohort of persons with TB–COVID-19 from a low TB–incidence setting (the United States) during the COVID-19 pandemic. Persons with TB and COVID-19 had overlapping sociodemographic and medical risk profiles known to be associated with each disease, including long-term care residence, diabetes, and end-stage renal disease. The frequency of death for persons with TB–COVID-19 was higher than persons with TB-only and depended on a shorter interval between TB and COVID-19 diagnoses (1 in 5 persons who had TB–COVID-19 co-diagnosed within 30 days died). However, COVID-19 was not independently associated with death among persons diagnosed with TB within 180 days when adjusted for age, underlying conditions, and TB disease severity, compared with those with 2020 TB-only patients. Among persons with TB–COVID-19, the timing of TB and COVID-19 co-diagnoses (i.e., within 90 days) remained a predictor of death, along with increasing age and being immunocompromised (non-HIV). Another analysis from California demonstrated an age-adjusted mortality rate ratio of 1.3 (95% CI 0.7‒2.5) for deaths among TB–COVID-19 patients compared with 2017‒2019 TB-only patients (*9*). That analysis did not adjust for underlying conditions (*9*). Increased mortality rates for persons with TB–COVID-19 has been repeatedly demonstrated in other settings when compared with persons with COVID-19 only (*5*–*8*). Other studies have demonstrated more severe COVID-19 disease classification among persons with TB–COVID-19 compared with persons diagnosed with COVID-19 without TB (*19*). However, few population-based studies have evaluated COVID-19 as a risk factor for all-cause mortality among persons with TB while adjusting for age, underlying conditions, and other potential confounders.

The baseline mortality rate for persons with TB was ≈9% annually in the United States in 2017 and 2018 (*18*). In our study, ≈17% of persons diagnosed with TB and COVID-19 within 180 days died. Nonetheless, in multivariable analysis corrected for age and underlying conditions, COVID-19 was not an independent predictor of death among persons with TB diagnosed within 180 days. Those findings suggest that poor outcomes for persons with TB–COVID-19 may be driven by the overlapping sociodemographic and medical risk factors common to each TB and COVID-19 that already place persons with TB at risk for death with TB, rather than the effect of COVID-19 coinfection alone. Compared with countries that have high TB prevalence, TB disease in the United States and other low TB incidence countries is more concentrated in older persons who have underlying conditions such as diabetes and renal disease (*18*). The timing of TB and COVID-19 co-diagnoses and its association with TB mortality warrants more investigation, given that our model demonstrated an association between a smaller diagnostic interval (90 days) and death among persons with TB–COVID-19. In addition to biological mechanisms to explain the association, persons with more severe COVID-19 may have been more likely to receive chest imaging and additional diagnostic testing to reveal concurrent TB.

Delayed TB diagnoses could have led to more severe TB disease at clinical evaluation in our analysis population. Another study from a low TB incidence setting showed a higher proportion of positive microscopic examinations during the COVID-19 pandemic compared with historical trends (*20*), similar to the observation in this US cohort of persons with TB–COVID-19. This finding suggests longer delays until TB diagnosis during the COVID-19 pandemic. The timing of TB diagnosis after COVID-19 (a substantial proportion had TB diagnosed within 14 days after COVID-19) could also reflect delayed TB diagnoses, suggesting that COVID-19 could have brought persons with undiagnosed TB into care.

TB program participation was nonrandom, which limits the representativeness of results to the entire United States, perhaps especially related to race and ethnicity. Nonetheless, the cohort represented a cross-section of US jurisdictions with varying TB prevalence. An important distinction in comparison with other studies is that we were unable to compare outcomes for persons with TB–COVID-19 with those for persons with COVID-19 only. Other limitations are that the completeness of COVID-19 case reporting may have differed by jurisdiction and phase of the epidemic. Missing data may have lessened the accuracy of some descriptive characteristics; missing death dates precluded hazards analyses of time to death. Longitudinal case management for persons on TB treatment probably captured most, but potentially not all, deaths among persons with TB. Our definition for disseminated TB is intended to capture most cases resulting from hematogenous spread that might be associated with delayed diagnoses or poor outcomes. It may not reflect all disseminated TB characterized by isolated extrapulmonary lymphadenitis or TB misclassified because of incomplete tissue sampling. The Bonferroni correction may have raised the risk for type II error in bivariate comparisons, and the small number of persons with TB–COVID-19 and having sociodemographic characteristics potentially influencing outcomes (e.g., experiencing homelessness) limited our ability to describe them. Strengths include the high completeness of sociodemographic data available in NTSS (*15*). Still, some underlying conditions strongly associated with poor COVID-19 outcomes (e.g., cardiovascular disease and obesity) were not available.

In conclusion, this analysis of a US cohort of persons with TB–COVID-19 suggests deaths among persons with TB–COVID-19 in the United States is concentrated in subgroups having shared characteristics known to increase risk for death with either disease alone. Timely consideration for TB disease among persons with COVID-19 and TB risk factors should be reinforced. Because death was associated with shorter intervals between co-diagnoses, prioritizing additional early medical interventions for persons with concurrent disease processes who are at highest risk for death might improve outcomes. COVID-19 patients with severe disease may be given immunomodulating treatments that could reactivate latent TB infection. Therefore, COVID-19 patients with risk factors for TB infection could be considered for screening and treatment of latent TB infection. Last, integration of screening for TB infection (risk factor review and serum interferon gamma release assays testing) with community COVID-19 prevention efforts among subpopulations with shared risk profiles, as has been done for persons at increased risk for COVID-19 and diabetes (*21*), may expand high-yield opportunities to prevent TB.

Appendix Additional information about characteristics and mortality of 333 persons with tuberculosis and COVID-19 in a cross-sectional sample from 25 jurisdictions, United States.
