# Supplementary material for: Characteristics of and Deaths among 333 Persons with Tuberculosis and COVID-19 in Cross-Sectional Sample from 25 Jurisdictions, United States
Source: Emerg Infect Dis. 2023 Oct;29(10):2016–23. doi: 10.3201/eid2910.230286 (PMC10521611; doi:10.3201/eid2910.230286)
Supplement: Appendix — Additional information about characteristics and mortality of 333 persons with tuberculosis and COVID-19 in a cross-sectional sample from 25 jurisdictions, United States. [file 23-0286-Techapp-s1.pdf]

Article DOI: https://doi.org/10.3201/eid2910.230286

EID cannot ensure accessibility for supplementary materials supplied by authors. Readers who have difficulty accessing supplementary content should contact the authors for assistance.

Characteristics of and Deaths among 333 Persons with Tuberculosis and COVID-19 in Cross-Sectional Sample from 25 Jurisdictions, United States

Appendix

Appendix Table 1. Methods used to identify 333 persons with TB/COVID-19 among 25 U.S. jurisdictions

Characteristic	no./N (%)
No. responding jurisdictions	26
Method for identifying persons with TB also diagnosed with COVID-19*	
With formal registry match using advanced software	*12/26 (46.2)*
Formal registry match only	10/26 (38.5)
Formal registry match and integrated data system	1/26 (3.8)
Formal registry match and manual search	1/26 (3.8)
Without formal registry match using advanced software	*14/26 (53.8)*
Integrated data system only	5/26 (19.2)
Manual search and integrated data system	3/26 (11.5)
Manual search only	6/26 (23.1)
Matching algorithm (among formal registry matches)	
Probabilistic	9/12 (75.0)
Deterministic	3/12 (25.0)
Software program (among formal registry matches)	
Project's SAS script	2/12 (16.6)
MatchPro	1/12 (8.3)
Link Plus	1/12 (8.3)
Locally developed program	5/12 (41.7)
Other‡	3/12 (25.0)
Data elements used to identify persons with TB/COVID-19 (among formal registry matches)†	
Name, birth date	3/12 (25.0)
Name, birth date, residence	5/12 (41.7)
Name, birth date, residence, sex	2/12 (16.7)
Name, birth date, race/ethnicity, sex	1/12 (8.3)
Name, birth date, residence, race/ethnicity, sex	1/12 (8.3)
Date through which COVID-19 registry was up-to-date at the time of match	
10/31/2021 or later	21/26 (80.8)
Before 10/31/2021§	3/26 (11.5)
Not reported	2/26 (7.7)

Legend: TB = tuberculosis; COVID-19 = coronavirus disease 2019.
* Integrated data system = name-based, person-centric surveillance database across disease conditions.
† All participants matched using first name, last name, and date of birth; other responses included phone number (2), surveillance system person ID (2) and social security number (1)
‡ Responses included WordCare/SQL (1), other SAS script (1), R (1)
§ Responses included 1/24/2021, 2/2/2021, and 8/31/2021

Appendix Table 2. Adjusted log-binomial association between COVID-19 (exposure) and all-cause death (outcome) among 4,091 persons with TB in 2020 by timing between TB and COVID-19 diagnoses, 24 U.S. jurisdictions

	Time between TB and COVID-19 diagnoses*								
	≤30 d	(N = 83 TB/COVID-19)		≤90 d	(N = 183 TB/COVID-19)		≤180 d	(N = 288 TB/COVID-19)	
Characteristic	no. (%) died	uPR (95% CI)	aPR (95% CI)	no. (%) died	uPR (95% CI)	aPR (95% CI)	no. (%) died	uPR (95% CI)	aPR (95% CI)
COVID-19 within timeframe									
No	450 (11.9)	Reference	Reference	450 (11.9)	Reference	Reference	450 (11.9)	Reference	Reference
Yes	17 (20.5)	1.7 (1.1–2.7)	1.2 (0.7–2.0)	39 (21.3)	1.8 (1.3–2.4)	1.3 (0.9–1.9)	48 (16.7)	1.4 (1.1–1.8)	1.0 (0.8–1.4)
Age									
<45 y	55 (3.3)	Reference	Reference	55 (3.2)	Reference	Reference	55 (3.2)	Reference	Reference
45–64 y	112 (9.6)	2.9 (2.1–4.0)	2.3 (1.6–3.4)	118 (9.9)	3.0 (2.2–4.1)	2.4 (1.7–3.5)	121 (9.9)	3.1 (2.3–4.2)	2.6 (1.8–3.7)
65–74 y	106 (19.5)	5.9 (4.3–8.0)	4.0 (2.7–5.9)	111 (19.7)	6.1 (4.4–8.2)	4.1 (2.8–6.0)	113 (19.5)	6.1 (4.5–8.4)	4.2 (2.9–6.1)
75–84 y	116 (32.5)	9.8 (7.2–13.2)	6.4 (4.3–9.4)	124 (33.4)	10.3 (7.6–13.8)	6.6 (4.5–9.7)	126 (33.2)	10.4 (7.8–14.1)	7.3 (5.1–10.6)
≥85 y	78 (50.3)	15.1 (11.2–20.5)	7.9 (5.1–12.1)	81 (51.3)	15.7 (11.6–21.3)	8.2 (5.4–12.5)	83 (51.2)	16.1 (12.0–21.8)	9.0 (6.1–13.4)
HIV status									
Negative	283 (8.7)	Reference	Reference	298 (8.9)	Reference	Reference	307 (8.9)	Reference	Reference
Positive	26 (16.5)	1.9 (1.3–2.7)	2.3 (1.4–3.8)	26 (16.2)	1.8 (1.3–2.6)	2.2 (1.3–3.7)	26 (15.5)	1.7 (1.2–2.5)	2.1 (1.3–3.5)
Missing/Unknown	158 (35.0)	4.0 (3.4–4.8)	2.2 (1.7–2.8)	165 (35.8)	4.0 (3.4–4.7)	2.2 (1.7–2.8)	165 (35.3)	4.0 (3.4–4.7)	2.1 (1.7–2.7)
End-stage renal disease									
No	403 (10.8)	Reference	Reference	422 (11.0)	Reference	Reference	431 (11.0)	Reference	Reference
Yes	64 (48.1)	4.5 (3.7–5.5)	2.1 (1.5–2.9)	67 (48.2)	4.4 (3.6–5.3)	2.1 (1.5–2.8)	67 (45.6)	4.2 (3.4–5.1)	1.8 (1.4–2.5)
Disseminated TB*									
No	354 (10.9)	Reference	Reference	372 (11.2)	Reference	Reference	381 (11.1)	Reference	Reference
Yes	113 (18.1)	1.7 (1.4–2.0)	1.5 (1.1–2.0)	117 (18.2)	1.6 (1.4–2.0)	1.5 (1.1–2.0)	117 (17.8)	1.6 (1.3–1.9)	1.5 (1.1–1.9)
Sputum smear status									
Negative	153 (8.5)	Reference	Reference	162 (8.8)	Reference	Reference	166 (8.8)	Reference	Reference
Positive	187 (11.8)	1.4 (1.1–1.7)	1.5 (1.1–1.9)	197 (12.1)	1.4 (1.1–1.7)	1.5 (1.1–1.9)	202 (12.1)	1.4 (1.1–1.7)	1.4 (1.1–1.8)
Missing/Unknown[†]	127 (25.3)	3.0 (2.4–3.7)	2.4 (1.7–3.2)	130 (25.3)	2.9 (2.3–3.5)	2.3 (1.7–3.1)	130 (24.8)	2.8 (2.3–3.5)	2.3 (1.8–3.1)

Legend: TB = tuberculosis; COVID-19 = coronavirus disease 2019; uPR = unadjusted prevalence ratio; aPR = adjusted prevalence ratio; CI = confidence interval
NTSS outcomes updated as of September 23, 2022. Five persons with TB/COVID-19 with unknown outcomes status. Puerto Rico and Los Angeles County excluded (remainder of California included) due to incompleteness of outcomes data.
* For persons diagnosed with both TB and COVID-19
[†] Disseminated TB = both pulmonary and extrapulmonary sites of disease, positive blood culture for Mtb, meningitis, or miliary pattern on imaging
[‡] Sputum smear status set to unknown for cases with extrapulmonary TB.
